# Dose tapering and treatment de-escalation of biologic therapies in chronic rhinosinusitis with nasal polyps: a narrative review

**DOI:** 10.3389/fsurg.2026.1933488

**Published:** 2026-08-18

**Authors:** Salma S. AlSharhan, Hussain J. Aljubran

**Affiliations:** 1 Department of Otolaryngology – Head and Neck Surgery, College of Medicine, Imam Abdulrahman Bin Faisal University, Dammam, Saudi Arabia; 2 Department of Otolaryngology – Head and Neck Surgery, Aljaber Ophthalmology and Otolaryngology Hospital, Alahsa Health Cluster, Alahsa, Saudi Arabia

**Keywords:** biologic therapy, chronic rhinosinusitis, CRSwNP, dose tapering, dupilumab, precision medicine, spacing

## Abstract

**Background:**

Biologic therapies have transformed the management of severe chronic rhinosinusitis with nasal polyps (CRSwNP), particularly in patients with uncontrolled type 2 inflammation. Although dupilumab, omalizumab, mepolizumab, benralizumab, tezepelumab, depemokimab, and stapokibart have demonstrated substantial clinical efficacy, the optimal duration and maintenance strategy for biologic therapy remain uncertain. The chronic nature of CRSwNP and the high cost of biologic treatment have stimulated growing interest in dose tapering and treatment de-escalation.

**Objective:**

To review the current evidence on biologic dose tapering in CRSwNP, summarize published tapering strategies, evaluate clinical outcomes, and discuss patient selection, monitoring, and future research priorities.

**Methods:**

A narrative review of the published literature on biologic dose tapering and treatment optimization in CRSwNP, including pivotal clinical trials, prospective observational studies, real-world cohorts, and current guideline recommendations.

**Results:**

Current evidence, derived predominantly from prospective observational studies and real-world cohorts, suggests that gradual extension of biologic dosing intervals can maintain disease control in carefully selected patients with sustained remission. Most studies reported preserved improvements in nasal polyp score, symptom burden, olfactory function, asthma control, and quality of life after extending treatment from every 2 weeks to every 4 weeks, with some patients successfully maintaining remission at 6-, 8-, 10-, or 12-week intervals. However, available evidence remains predominantly observational, and tapering protocols, eligibility criteria, and definitions of remission are heterogeneous.

**Conclusion:**

Biologic dose tapering represents a promising treatment optimization strategy for carefully selected patients with stable CRSwNP, particularly those receiving dupilumab. However, standardized tapering protocols and high-quality randomized controlled trials are required before routine implementation. Future research should focus on biomarker-guided patient selection, long-term clinical outcomes, and the cost-effectiveness of biologic dose optimization.

## 1 Introduction

Chronic rhinosinusitis (CRS) is a heterogeneous inflammatory disorder of the sinonasal mucosa characterized by persistent symptoms lasting more than 12 weeks. Affecting approximately 5%–12% of adults worldwide, CRS is associated with substantial healthcare utilization, impaired quality of life, reduced productivity, and considerable socioeconomic burden. Chronic rhinosinusitis with nasal polyps (CRSwNP) represents a severe disease phenotype characterized by diffuse mucosal inflammation, recurrent polyp formation, and persistent sinonasal dysfunction (1–3).

The predominant inflammatory endotype of severe CRSwNP is type 2 inflammation, characterized by eosinophilic infiltration and increased expression of interleukin (IL)-4, IL-5, IL-13, immunoglobulin E (IgE), and other inflammatory mediators. This endotype is frequently associated with asthma, nonsteroidal anti-inflammatory drug-exacerbated respiratory disease (N-ERD), and allergic rhinitis, supporting the concept of united airway disease (2–6).

Conventional management includes intranasal corticosteroids, short courses of systemic corticosteroids, and endoscopic sinus surgery (ESS). Despite these interventions, a substantial proportion of patients experience persistent or recurrent disease. Repeated systemic corticosteroid exposure is associated with significant adverse effects, while revision surgery carries cumulative risks and does not adequately address the underlying inflammatory process. Consequently, achieving durable disease control while minimizing treatment-related morbidity has become a major therapeutic goal in severe CRSwNP (2, 7–9).

The introduction of biologic therapies has transformed the treatment paradigm for severe uncontrolled CRSwNP. By selectively targeting key mediators of type 2 inflammation, biologics provide disease control through targeted immunomodulation rather than nonspecific anti-inflammatory effects. Dupilumab, a monoclonal antibody directed against the IL-4 receptor *α* subunit, inhibits both IL-4 and IL-13 signaling and was the first biologic approved for CRSwNP. Pivotal clinical trials demonstrated significant improvements in nasal polyp size, symptom severity, olfactory function, health-related quality of life, and the need for systemic corticosteroids and revision surgery. Subsequently, omalizumab, mepolizumab, benralizumab, tezepelumab, depemokimab, and stapokibart have expanded the therapeutic options available for patients with type 2 inflammatory disease (10–20).

Current biologic regimens are based on fixed dosing schedules established in pivotal randomized clinical trials. For dupilumab, the approved maintenance regimen is 300 mg every two weeks (Q2W), as evaluated in the SINUS-24 and SINUS-52 trials. Although these studies established the efficacy and safety of continuous therapy, they were not designed to determine the minimum effective maintenance dose or the optimal duration of treatment (7). As biologic use has expanded, the need for long-term treatment, high healthcare costs, treatment burden, and the goal of minimizing unnecessary drug exposure have prompted increasing interest in biologic dose tapering and interval extension after sustained clinical remission (21–24).

Unlike treatment discontinuation, dose tapering aims to maintain disease control while reducing cumulative biologic exposure. Over the past several years, emerging real-world evidence has demonstrated that selected patients with durable remission can maintain disease control after extending dupilumab dosing intervals (7, 18–20). These findings suggest that biologic treatment intensity may be safely reduced during the maintenance phase in carefully selected patients without compromising clinical outcomes.

Despite these encouraging observations, several important questions remain unanswered. Current evidence is derived predominantly from observational studies, tapering protocols and eligibility criteria are heterogeneous, definitions of remission are inconsistent, and validated biomarkers to guide treatment optimization are lacking. Consequently, current international guidelines have not yet incorporated standardized recommendations for biologic dose tapering or treatment de-escalation.

This narrative review summarizes the current evidence on biologic dose tapering in CRSwNP, reviews real-world outcomes findings, outlines important rationale factors related to dose tapering, including practical approaches to patient selection and monitoring, and concludes by summarizing evidence-based guidelines, current gaps, and future research directions.

## 2 Methods

This narrative review was conducted to summarize the current evidence regarding biologic dose tapering and treatment de-escalation in CRSwNP. A comprehensive literature search was performed using PubMed/MEDLINE, Embase, and Google Scholar to identify relevant publications from database inception through May 2026. The search strategy included combinations of the following keywords and Medical Subject Headings (MeSH) terms (individually or in association): “Chronic rhinosinusitis with nasal polyps”, “CRSwNP”, “biologics”, “dupilumab”, “omalizumab”, “mepolizumab”, “benralizumab”, “tezepelumab”, “depemokimab”, “stapokibart”, “dose tapering”, “dose reduction”, “interval extension”, “de-escalation”, “treatment optimization”, and “maintenance therapy”.

Randomized controlled trials, prospective and real-world cohort studies, systematic and narrative reviews, and international guideline documents published in English were included. Reference lists of eligible articles were also manually screened for additional studies. Evidence on biologic dose reduction, interval extension, treatment optimization, and maintenance strategies in CRSwNP was prioritized. Given the limited CRSwNP-specific data, relevant evidence from other type 2 inflammatory and immune-mediated diseases, including asthma, atopic dermatitis, psoriasis, rheumatoid arthritis, and inflammatory bowel disease, was also reviewed to provide biological and clinical context. The available evidence was synthesized to summarize current knowledge, identify evidence gaps, and highlight future research priorities. The review was conducted according to the Scale for the Assessment of Narrative Review Articles (SANRA) principles to enhance methodological rigor (25).

## 3 Rationale for biologic dose tapering and treatment optimization

Biologic therapies have transformed the management of CRSwNP, shifting treatment goals from symptom control to sustained disease remission. However, pivotal clinical trials established continuous maintenance dosing without defining the minimum treatment needed to maintain remission (13–17). Consequently, biologic dose tapering has emerged as a potential strategy to optimize long-term therapy while reducing unnecessary drug exposure (18–20).

Biologic therapies selectively target the principal mediators of type 2 inflammation: dupilumab and stapokibart inhibit IL-4 and IL-13 signaling through blockade of the IL-4 receptor *α* subunit; mepolizumab, depemokimab, and benralizumab target the IL-5 pathway; omalizumab neutralizes IgE; and tezepelumab blocks thymic stromal lymphopoietin (10–16). By suppressing the underlying inflammatory cascade, these agents reduce eosinophilic inflammation, improve epithelial barrier function, and attenuate tissue remodeling. Clinical improvement with dupilumab is observed early after treatment initiation and continues throughout the first year of therapy, suggesting progressive restoration of sinonasal immune homeostasis. Once sustained disease control has been achieved, maintaining remission may require less intensive biologic exposure than during the induction phase (10–13).

The pharmacokinetic characteristics of monoclonal antibodies further support interval extension as a maintenance strategy. Their prolonged half-life and sustained receptor occupancy permit continued suppression of type 2 inflammatory pathways beyond the standard dosing interval. Importantly, dose tapering should be distinguished from treatment discontinuation. In the SINUS-24 trial, complete withdrawal of dupilumab resulted in gradual disease recurrence, whereas interval extension preserves ongoing biologic activity while reducing treatment intensity (7). Similar de-escalation strategies have been successfully implemented in other chronic immune-mediated diseases, including rheumatoid arthritis, psoriasis, axial spondyloarthritis, and atopic dermatitis, providing additional support for treatment optimization in CRSwNP (26–30).

Beyond its biological rationale, dose tapering addresses several practical challenges associated with long-term biologic therapy. CRSwNP is a chronic relapsing disease, and many patients require prolonged or even indefinite treatment to maintain disease control. Maintaining Q2W dosing throughout this period may not be necessary for patients who achieve maintenance of disease remission. Furthermore, biologics represent one of the most expensive treatment modalities for CRSwNP, and even modest reductions in cumulative drug exposure could substantially improve healthcare sustainability without compromising clinical outcomes (31). Less frequent dosing may also reduce treatment burden, improve convenience and adherence, and enhance patient satisfaction while potentially limiting cumulative treatment-related adverse events, although long-term safety data remain limited (18–20).

## 4 Current evidence on biologic dose tapering in CRSwNP

Evidence supporting biologic dose tapering in CRSwNP has expanded considerably in recent years. Because pivotal randomized clinical trials were designed to establish the efficacy and safety of continuous biologic therapy rather than treatment optimization, current evidence is derived predominantly from prospective observational studies, real-world cohorts, and multicenter registries. To date, nearly all published data involve dupilumab because of its earlier regulatory approval and broader clinical use (Tables 1, 2) (7, 18–20, 32–41).

**Table 1:** Published studies evaluating the feasibility of biological dose tapering in CRSwNP.

Study	Country	Study design	Sample size	Biological agent	Patients started tapering	Tapering strategy	Successful rate of tapering	Successful criteria	Limitations
Fujieda et al. (35)	Japan	Randomized control trial	23	Dupilumab (300 mg)	3	Q2W → Q4W	100%	≥1 point improvement in NPS	Small sample size with limited conclusions about tapering
Bachert et al. (7)	Multinational	Randomized control trial	295	Dupilumab (300 mg)	145	Q2W → Q4W	NR	NR	Interval change was protocol-defined; limited conclusion about tapering
De Corso et al. (20)	Italy	Prospective cohort	926	Dupilumab (300 mg)	173	Q2W → Q4W	NR	Excellent response on EPOS/EUFOREA criteria	Remission definitions were not externally validated; heterogeneous reasons for interval extension
Elzinga et al. (19)	Netherlands	Prospective cohort	202	Dupilumab (300 mg)	189	Q2W → Q4W → Q6W → Q8W → Q12W	55%	Excellent response on EPOS/EUFOREA criteria	N-ERD diagnosis was largely history-based; no blinded or randomized comparison group
De Corso et al. (20)	Italy	Prospective cohort	148	Dupilumab (300 mg)	71	Q2W → Q4W	79%	Excellent response on EPOS/EUFOREA criteria	Remission definitions were not externally validated; heterogeneous reasons for interval extension
van der Lans et al. (18)	Netherlands	Prospective cohort	228	Dupilumab (300 mg)	228	Q2W → Q4W → Q6W → Q8W	94%	Excellent response on EPOS/EUFOREA criteria	Declining sample size at later follow-up; no blinded or randomized comparison group
Campion et al. (36)	Austria	Retrospective cohort	224	Dupilumab (300 mg)	90	Q2W → Q4W → Q6W	89%	NR	Retrospective single-center cohort; interval extension was voluntary and performed after stable control
Habenbacher et al. (37)	Austria	Retrospective cohort	35	Dupilumab (300 mg)	35	Q2W → Q4W → Q6W → Q8W → Q10W	77%	Excellent response on EPOS/EUFOREA criteria	Retrospective single-center design with small sample size; non-randomized comparison group
Nakashima et al. (32)	Japan	Retrospective cohort	107	Dupilumab (300 mg)	61	Q2W → Q3W → Q4W	57%	Excellent response on EPOS/EUFOREA criteria	Dosing-interval extension was not the primary study question, limiting applicability to de-escalation strategies
D’Ascanio et al. (39)	Italy	Retrospective cohort	42	Dupilumab (300 mg)	42	Q2W → Q4W	71%	Excellent response on EPOS/EUFOREA criteria	Small single-center observational letter; limited statistical power
Alghamdi et al. (40)	Saudi Arabia	Retrospective cohort	42	Dupilumab (300 mg)	42	Q2W → Q4W	93%	Excellent response on EPOS/EUFOREA criteria	Retrospective single-cohort study from one tertiary center; small sample size; no randomized Q2W control group
Kang et al. (34)	Korea	Retrospective cohort	36	Dupilumab (300 mg)	36	Q2W → Q4W	72%	Excellent response on EPOS/EUFOREA criteria	Retrospective monthly-dosing study driven largely by cost and affordability rather than formal response-guided tapering
Appel et al. (38)	Germany	Retrospective cohort	29	Dupilumab (300 mg)	29	Q2W → Q4W → Q6W	100%	NR	Small retrospective single-center cohort; timing of tapering was influenced by follow-up delays and insurance approval.
Martines and Zamorano (33)	Spain	Retrospective case series	5	Dupilumab (300 mg)	5	Q2W → Q4W	100%	NR	Very small case series of five patients with limited follow-up period; no control group

**Table 2:** Comparison of published biologic dose-tapering protocols in CRSwNP.

Study	Criteria for start of tapering	Inter-dose interval	Maximum interval achieved	Follow-up	Key findings
Fujieda et al. (35)	Improvement of ≥2 points in the Nasal Polyp Score	6 months	Q4W	13 months	Tapering to dupilumab every 4 weeks was associated with maintained CRSwNP outcomes in selected stable patients compared with those who remained on the standard dose regimen.
Bachert et al. (7)	Not applicable (randomized)	6 months	Q4W	6 months	Monthly dosing maintained substantial clinical benefit compared with continuous biweekly dosing, although objective outcomes were generally numerically stronger with continuous Q2W dosing. However, the study was not designed as a tapering trial.
De Corso et al. (41)	Based on the presence of side effects, patient preference, and physician assessment	At least 6 months	Q4W	24 months	Dupilumab was associated with sustained improvements in polyp burden, symptoms, quality of life, and olfaction over long-term follow-up, while extending the dosing interval was not associated with compromised outcomes in selected patients and continued treatment benefited even late responders.
Elzinga et al. (19)	Moderate to excellent response based on EPOS criteria	At least 6 months	Q12W	24 months	Tapering appeared feasible even in the presence of N-ERD. Most patients-maintained remission despite progressive interval extension; the severe inflammatory phenotype remained generally well controlled.
De Corso et al. (20)	Based on the presence of side effects, patient preference, and physician assessment	At least 6 months	Q4W	24 months	The tapering group who extended to monthly dosing showed comparable disease control and remission over 2 years to the standard dosing group. Also, asthma control test results were comparable between the two groups.
van der Lans et al. (18)	Moderate to excellent response based on EPOS criteria	At least 6 months	Q8W	24 months	Clinical improvement was generally maintained over 2 years in most patients; tapering was possible up to at least Q8W in selected responders.
Campion et al. (36)	NR	15 months	Q6W	36 months	Patients with CRSwNP showed greater improvement in symptoms during the first year after dupilumab tapering compared with those on Q2W dosing; however, this improvement was not statistically significant after the first year, and both groups remained comparable. The study also suggests that increasing the injection interval may alleviate treatment-related side effects in some patients without clear loss of efficacy.
Habenbacher et al. (37)	Moderate to excellent response based on EPOS criteria after 12 months of Q2W dose	At least 6 months	Q10W	15 months	Dupilumab tapering was associated with maintained CRSwNP control in selected patients, although some required re-escalation to Q2W and others could not tolerate Q4W but remained controlled on Q3W.
Nakashima et al. (32)	Moderate to excellent response based on EPOS criteria	NR	Q4W	12 months	Half of the patients tolerated extension of the dupilumab dosing interval to every 3–4 weeks, and this spacing was not associated with worsening asthma control, while clinical outcomes at 12 months remained similar or better than earlier treatment points.
D’Ascanio et al. (39)	Excellent response based on EPOS criteria	12 months	Q4W	24 months	Less frequent dosing was associated with maintained symptomatic relief and objective disease control, with sustained improvements in nasal polyp burden and SNOT-22 quality-of-life scores and no significant difference compared with patients who continued standard dosing.
Alghamdi et al. (40)	Moderate to excellent response based on EPOS criteria	12 months	Q4W	24 months	Extending dupilumab from biweekly to monthly dosing was associated with maintained symptom control in most clinically stable patients with CRSwNP, including those with comorbid asthma.
Kang et al. (34)	NR	NR	Q4W	6 months	Although this study was not a formal “tapering-after-control” study, monthly dupilumab dosing was associated with significant 6-month improvements in CRSwNP outcomes, suggesting that a Q4W interval may provide meaningful efficacy and safety in selected real-world patients.
Appel et al. (38)	Patient desire with NPS ≤1 per side, improved SNOT-22, no need for steroid, and improved lower airway disease	13–23 months	Q6W	NR	Individualized interval extension was associated with maintained CRSwNP control, with no reported recurrence of nasal polyps or sinonasal symptoms and no worsening in quality of life, smell, or asthma control.
Martines and Zamorano (33)	NR	24 months	Q4W	6 months	Extending the dosing interval to every 4 weeks in patients with severe eosinophilic asthma and CRSwNP was associated with maintained disease control over 6 months, with asthma control test and SNOT-22 scores improving or remaining stable.

### 4.1 Evolution of the evidence

The first indication that biologic dose reduction might be feasible originated from the SINUS-52 trial, in which patients transitioned from Q2W to every 4 weeks (Q4W) dupilumab after 6 months while maintaining clinical benefit (7). Although this study was not designed to evaluate dose tapering, it provided the initial proof-of-concept that reduced treatment intensity could preserve disease control. Subsequent prospective observational studies have specifically investigated interval extension in patients who achieved sustained control of sinonasal inflammation (7, 18–20, 32–41).

### 4.2 Evidence from prospective and real-world studies

Across the published literature, a generally consistent pattern has emerged. Patients with sustained disease control following 6–24 months of standard dupilumab therapy generally maintained improvements in both subjective and objective outcomes. These outcomes included nasal polyp score (NPS), Sino-Nasal Outcome Test-22 (SNOT-22), olfactory function, and overall health-related quality of life after gradual extension of dosing intervals. Furthermore, related laboratory markers remained stable in most of the studies (including eosinophil counts and total IgE), indicating continued suppression of type 2 inflammation (18–20, 32–41). Similarly, control of comorbid lower-airway disease was measured in some of the studies with maintained outcomes in the majority of patients (using asthma control test, lung function, and other subjective outcomes). However, a small proportion of patients had to return to their standard dose, due to failure to maintain control of CRSwNP or coexisting lower-airway disease (18, 20, 33, 36, 40).

Extension from Q2W to Q4W represents the most frequently studied and consistently successful tapering strategy. In carefully selected patients, interval extension has subsequently been prolonged to a longer interval (18, 19, 36–38). Collectively, these findings suggest that biologic treatment intensity can be safely de-escalated during the maintenance phase in appropriately selected patients.

One of the reported indications of dupilumab was persistent peripheral eosinophilia (20, 41). Dupilumab-associated hypereosinophilia is thought to result from reduced eosinophil migration from the circulation into tissues following IL-4 and IL-13 pathway blockade (42–44). Hence, dupilumab tapering may theoretically reduce cumulative pharmacologic exposure, which could help in normalizing eosinophil levels in selected patients. Supporting this theory, some studies reported a modest decline in blood eosinophils after transition to Q4W interval, while disease and asthma control remained stable (20, 33, 40, 41). However, the current evidence is observational and does not establish tapering as a definitive treatment for dupilumab-induced hypereosinophilia but could offer a possible option in some patients with individualized decisions.

Overall, the available studies suggest that dupilumab interval extension is generally safe in carefully selected patients with stable CRSwNP and does not appear to increase adverse events compared with continued Q2W treatment. For example, Campion et al. reported fewer dupilumab-related adverse events after interval extension, suggesting that reduced cumulative exposure may lessen complaints (36). However, because most evidence is observational and tapering was offered mainly to stable responders, it cannot yet be concluded that interval extension directly reduces adverse events.

### 4.3 Limitations of the current evidence

The current evidence base remains limited. Most published studies are observational and involve relatively small patient populations. Standardized definitions of clinical remission and treatment success are lacking, tapering protocols remain heterogeneous, and validated clinical or molecular biomarkers capable of predicting successful dose reduction have not yet been identified. Moreover, the current evidence is largely limited to dupilumab tapering, and the use of tapering strategies on other biological agents is lacking. Therefore, future studies are still needed to assess whether similar tapering strategies on other biological agents in well-controlled CRSwNP patients can be safely applied. Additionally, the high successful rates of excellent responders reported in the current literature may overestimate the real-world success of tapering, since studies reporting successful dupilumab interval extension may be more likely to be published than experiences in which tapering failed. Although most of the included studies showed high success rates for dupilumab tapering, these findings should not be interpreted as evidence that tapering is suitable for all dupilumab-treated patients. In fact, most of the patients who were included in these studies for dose spacing were excellent responders to the standard dose interval, creating an inherent selection toward favorable outcomes. Nevertheless, this limitation highlights that biologic dose tapering should currently be regarded as an individualized treatment optimization strategy for well-controlled patients.

### 4.4 Practical strategies for biologic dose tapering

**Figure 1:**
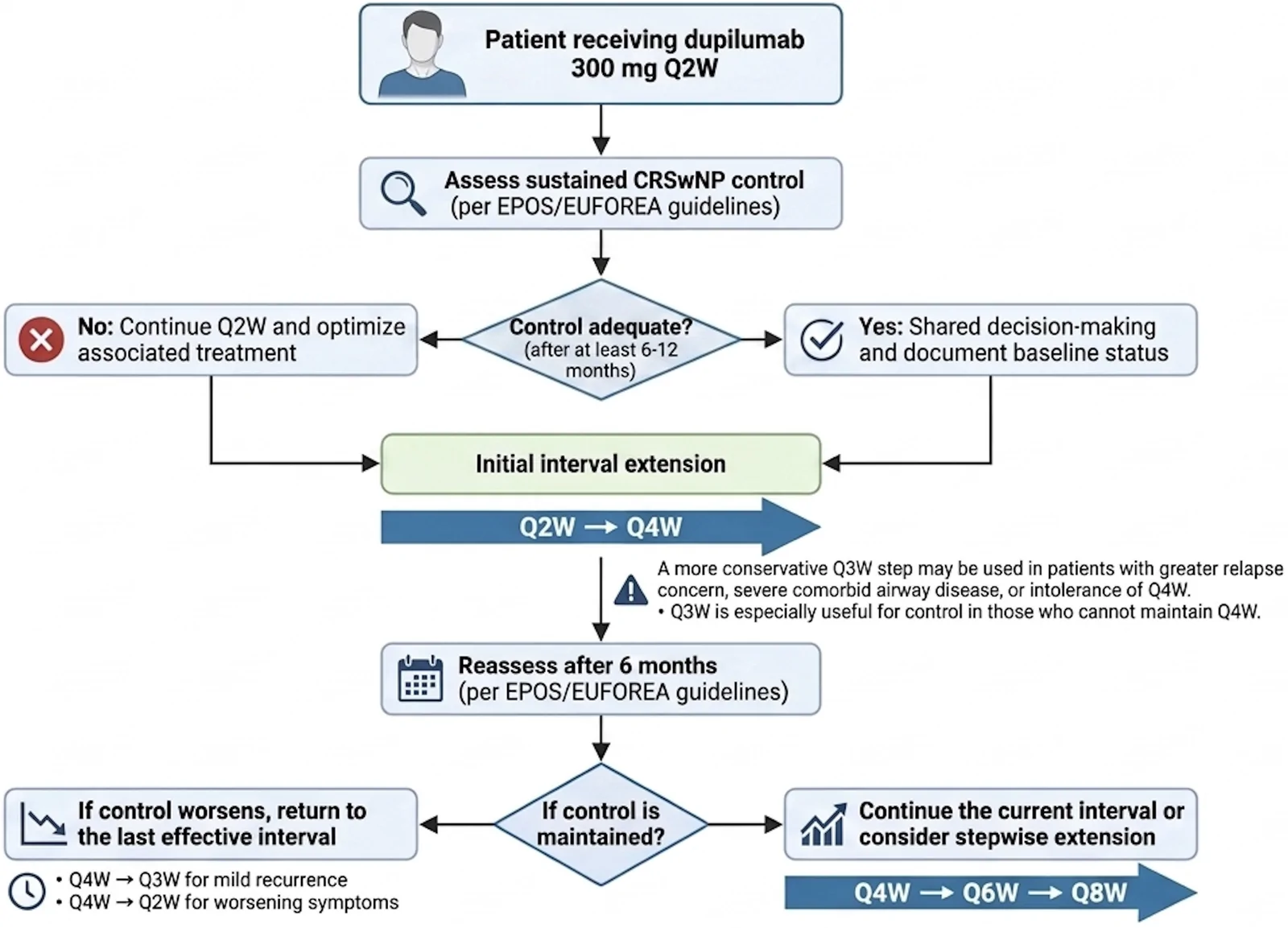
Proposed clinical pathway for dupilumab dosing-interval extension in patients with CRSwNP. CRSwNP, chronic rhinosinusitis with nasal polyps; Q2W, every 2 weeks; Q3W, every 3 weeks; Q4W, every 4 weeks; Q6W, every 6 weeks; Q8W, every 8 weeks.

Although biologic dose tapering has gained increasing attention in recent years, no universally accepted protocol currently exists for patients with CRSwNP. Published studies differ with respect to the timing of tapering, eligibility criteria, interval-extension schedules, monitoring strategies, and definitions of treatment success. Nevertheless, several common principles have emerged that may help guide clinical practice. Figure 1 presents a proposed evidence-informed framework for biologic tapering in CRSwNP.

### 4.5 Patient selection

Based on current evidence, interval extension should be considered only in patients who have achieved durable suppression of disease activity after an adequate period of standard biologic therapy. Most of the studies have used the EPOS/EUFOREA criteria to demonstrate stable sinonasal symptoms, with marked reduction of nasal polyp size, meaningful improvement in SNOT-22 and sinonasal symptoms, improved sense of smell, no recent need for systemic corticosteroids, and adequate enhancement of asthma or other type 2 comorbidities. In this criterion, the overall response is categorized into no response (0 criteria met), poor to moderate response (1–3 criteria met), and good to excellent response (4–5 criteria met) (45). Hence, most of the studies that demonstrated the outcomes of tapering have used the EPOS/EUFOREA criteria to define those patients who are eligible for tapering with sustained moderate to excellent response (18, 19, 32, 37, 39, 40). Conversely, few other studies have used different path to define those who are eligible for tapering (such as the improvement of at least 2 points in the NPS) (35). However, the decision to extend the dosing interval should be individualized and made jointly with the patient after discussing the potential benefits and risks.

Careful patient selection is fundamental to successful biologic dose tapering. Based on the current evidence, failure of biological tapering was not uniformly reported across studies and was often reported indirectly through patients who were return to shorter dosing intervals. For instance, Alghamdi et al. reported that three patients in their cohort returned to their regular interval due to worsening asthma control despite maintained sinonasal control (40). Another study reported that patient who failed dose spacing were more likely to have N-ERD, suggesting that patients with greater type 2 inflammatory burden or complex united-airway disease may require closer monitoring during tapering (36). Hence, biological tapering may be less suitable for patients with poor or moderate response, recurrent polyp growth, recent systemic corticosteroid use, recent rescue surgery, uncontrolled asthma, frequent lower-airway exacerbations, or inability to comply with close follow-up.

### 4.6 Timing and interval extension

The optimal timing for dose tapering has not yet been established. According to the EPOS/EUFOREA guideline, it was recommended to have two initial evaluation periods after the initiation of a new biological therapy (after 6 months and after 12 months), followed by annual assessment to evaluate the patients’ response (45). Hence, most of the studies have followed this criterion with at least 6–12 months of controlled symptoms before tapering, although longer periods of stability were used in some real-world cohorts (18, 19, 32, 37, 39, 40). In the SINUS-52 trial, patients transitioned from Q2W to Q4W dupilumab after 6 months irrespective of clinical response (7). However, most real-world studies adopted a more conservative approach, in which the patient must be excellent responder before tapering, with no fixed period.

A gradual, stepwise approach is consistently favored over abrupt reductions in treatment intensity. Tapering should be performed by maintaining the 300 mg dose and progressively extending the interval between injections. The best-supported initial step is from Q2W to Q4W, while Q3W may be used as a more conservative intermediate schedule in patients with a higher risk of relapse or in those who do not maintain control on Q4W (32). In selected individuals with sustained remission, treatment intervals have subsequently been prolonged to six-week and eight-week intervals while maintaining clinical remission (18, 19, 36–38). Longer intervals were demonstrated in few studies, though it was applied on very few patients and should be reserved for highly stable individuals under close specialist supervision (18, 37). Overall, biologic dose tapering should be viewed as an extension of precision medicine aimed at identifying the minimum biologic exposure required to sustain remission.

### 4.7 Monitoring during dose reduction

Regular clinical monitoring is essential throughout biologic dose tapering to ensure sustained disease control and allow early recognition of relapse. Follow-up must be performed at least every 6 months, including both objective and patient-reported outcome measures based on EPOS/EUFOREA guideline. Moreover, it is always important to maintain the coexisting lower-airway disease, if present (45).

### 4.8 Loss of response and re-attempt

Biologic dose tapering should be individualized and implemented using a gradual, reversible approach. Patients who maintain stable disease control after interval extension may undergo further prolongation of dosing intervals, whereas recurrence of symptoms, worsening endoscopic findings, deterioration in olfactory function, or loss of asthma control should prompt re-escalation to the previous effective dosing schedule. Based on the available evidence, biologic dose tapering showed wide range of successful rates ranging between 55%–100% based on the previous studies (18–20, 32–41). However, many factors could affect the outcomes of extending dose interval. Therefore, loss of control should be identified through a combination of patient-reported and objective findings rather than a single measure. EPOS/EUFOREA poor-response criteria may help structure the assessment, although standardized definitions of tapering failure have not yet been validated. When disease control deteriorates, that does not necessarily exclude future dose tapering trials. Once disease control has been re-established and maintained on the previous effective dosing regimen, tapering re-attempt may be considered using smaller interval changes, a longer stabilization period, and closer follow-up. For instance, a patient who relapsed on Q4W may later tolerate Q3W, but repeated tapering should remain individualized because evidence regarding the optimal timing and success of second attempts is still limited (37).

### 4.9 Regulatory considerations

Currently, the Food and Drug Administration (FDA) has approved three drugs for the treatment of CRSwNP, including dupilumab, omalizumab, and mepolizumab (41). However, differences in regional and country-specific regulations can affect the application of biological dosing strategies in treating CRSwNP. Worldwide, the use of alternative dosing schedules may be restricted in healthcare systems when they fall outside the approved product label. For instance, the routine interval of dupilumab in Europe and the United States is approved with Q2W interval, and currently extending this regimen is not explicitly established as standard labelled dosing in these countries (41, 45). However, in some countries, including Japan, extension of the dupilumab dosing interval to Q4W has been approved as a treatment option for CRSwNP (32). Therefore, regional regulatory and reimbursement considerations should be considered when adjusting biologic dosing intervals.

### 4.10 Tapering operationalization in practice

Extending dupilumab intervals in practice should be planned through shared decision-making that weighs lower treatment burden, cost, and adverse effects against relapse risk. Shared decision-making was defined ‘an approach where clinicians and patients make decisions together using the best available evidence (46, 47). Thus, a clear discussion with the patient is essential to ensure a proper understanding of the benefits of increasing the interval between doses, while acknowledging the possibility of worsening CRSwNP symptom control. In patients who fail to tolerate tapering and experience persistent symptom relapse, the dosing interval should be shortened to the previous effective regimen and may require a return to the standard dose. However, failure of the first tapering attempt does not always preclude future reattempts in selected patients. A summary of the suggested stage-based operational pathway, based on the currently available evidence, is presented in Table 3.

**Table 3:** Stage-based operational pathway for implementing dupilumab interval extension in CRSwNP.

Stage	Clinical practice point
Identifying potential responders	Consider tapering only after clear and sustained CRSwNP control, preferably after at least 6–12 months of response on standard dupilumab; suitable patients should have good to excellent response based on the EPOS/EUFOREA guidelines.
Decision-making	Use shared decision-making, especially because many interval-extension regimens are off-label or not yet standardized; discuss expected benefits such as lower treatment burden, fewer injections, lower cost, and possibly fewer adverse effects, balanced against relapse risk.
Operationalization	A practical CRSwNP pathway is Q2W → Q4W, reassess after a stable period, then consider longer intervals such as Q6W then Q8W only in highly stable patients.
Timing between tapering steps	At least 3–6 months between spacing steps, with 6 months being safer and more consistent with several CRSwNP tapering protocols.
Monitoring	Review the patient every six months and assess symptoms, endoscopic polyp burden, smell, need for systemic corticosteroids or surgery, and asthma control when applicable.
Loss of response	The response criteria of EPOS/EUFOREA guidelines can be used, in which poor response criteria is considered for tapering failure.
Re-escalation	Return to the last effective interval when CRSwNP symptoms control worsens; patients deteriorating on Q4W may be stepped back to Q3W, while more substantial sinonasal or lower-airway relapse should prompt return to Q2W.
Re-attempt	Once control has been re-established, tapering may be attempted again later using smaller interval changes, longer stabilization periods, and closer clinical follow-up.

### 4.11 Tapering cost-effectiveness

Biologic treatment has significantly reduced the clinical burden in patients with CRSwNP and the need for revision ESS. Nevertheless, biologics are known for their high long-term cost compared with conventional treatment for CRSwNP (48). In a cost-utility analysis, Scangas et al. reported that the cost of dupilumab could reach $536,420, compared with $50,436 for conventional treatment. Moreover, the annual cost of dupilumab would need to be reduced to $855 to match the cost-effectiveness of conventional treatment (49). Despite the limited evidence on tapering cost-effectiveness, extending the dupilumab dosing interval may help achieve a more affordable cost, allowing more patients to benefit from this therapy. However, future studies are still needed to comprehensively evaluate cost-effectiveness of dose spacing across different reimbursement models, such as drug costs, reduce systematic corticosteroid use, surgery avoidance, and adverse events.

## 5 Future directions

Although emerging real-world evidence suggests that biologic dose tapering is feasible in selected patients with well-controlled CRSwNP, several important questions remain unresolved. Prospective randomized controlled trials comparing continuous maintenance therapy with dose-tapering strategies are needed to establish the optimal timing for tapering, define standardized interval-extension protocols, and determine the long-term durability of disease remission.

Future research should also focus on identifying reliable clinical, endoscopic, radiologic, and molecular biomarkers that predict successful dose reduction and sustained remission. Integrating inflammatory biomarkers with disease endotypes may enable individualized treatment optimization and further advance precision medicine in CRSwNP. In addition, robust health-economic analyses are needed to evaluate the cost-effectiveness of biologic dose optimization across different healthcare systems. Finally, further studies should determine whether tapering strategies can be safely and effectively extended to biologics targeting IL-5, the IL-5 receptor, and IgE, thereby broadening the applicability of treatment optimization across the full spectrum of biologic therapies. For instance, mepolizumab intervals were successfully extended in one of the studies in patients with severe eosinophilic asthma or eosinophilic granulomatosis with polyangiitis (50, 51). Similar evidence regarding extending mepolizumab injection spacing in CRSwNP remain limited.

## 6 Conclusion

Biologic therapies have transformed the management of severe CRSwNP, providing sustained control of type 2 inflammation and substantial improvements in patient outcomes. Emerging real-world evidence suggests that gradual extension of dosing intervals, particularly with dupilumab, can maintain disease control in carefully selected patients with sustained remission while reducing treatment burden and healthcare costs. However, current evidence remains predominantly observational, and standardized tapering protocols have yet to be established. Hence, future randomized control trials and biomarker-driven approaches will be essential to establish evidence-based tapering strategies and integrate biologic dose optimization into routine clinical practice.
